# Orientational Switching as an Extra Degree of Freedom in Self-Assembled C_70_ and Octanethiol on Au(111)

**DOI:** 10.1021/acs.langmuir.6c01243

**Published:** 2026-06-03

**Authors:** Ying Gao, Bosheng Li, Hualin Yang, Haoxuan Ding, Jianzhi Gao, Minghu Pan, Quanmin Guo

**Affiliations:** † School of Physics and Astronomy, 1724 University of Birmingham, Birmingham B15 2TT, U.K.; ‡ School of Materials Science and Engineering, 12465 Peking University, Beijing 100871, People’s Republic of China; § School of Physics and Information Technology, 12401 Shaanxi Normal University, Xi’an 710119, People’s Republic of China

## Abstract

We investigated the coassembly of oval-shaped C_70_ and rod-like octanethiol on the Au(111) substrate. We find that C_70_ can switch between two orientations: (i) long axis perpendicular to the substrate and (ii) long axis parallel to the substrate. This switching capability offers an extra degree of freedom in optimizing space filling, and it leads to structural diversity. By switching between two orientations, the C_70_ molecule effectively plays a dual role in the creation of the self-assembled structures. The orientation of the octanethiol rod can also change in response to the local environment. Such an orientational degree of freedom is expected to play an important role in many physical systems covering multiple length scales.

## Graphical abstract

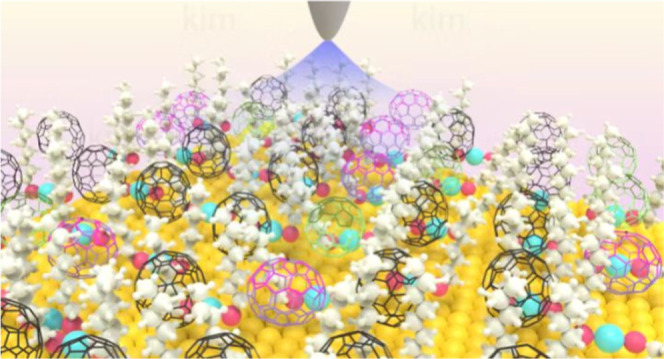

## Introduction

Molecular self-assembly at solid surfaces has been widely studied as a bottom-up strategy for constructing ordered nanoscale structures.^1–6^ Molecular orientation is known to influence packing and symmetry in two-dimensional assemblies, and its role has been extensively discussed in previous studies.^6,7^ In recent years, increasing attention has also been devoted to multicomponent molecular assemblies at solid interfaces, where different molecular species can coexist and interact cooperatively to generate structures with higher complexity than those formed by single-component systems.^8–13^ These studies have shown that multicomponent assembly can provide access to diverse architectures, including host–guest networks, bicomponent and multicomponent supramolecular structures, and hierarchical surface-confined patterns.^10–12,14^ However, the presence of multiple molecular species also introduces additional complexity because the individual components may differ in adsorption strength, mobility, intermolecular interactions, and response to the substrate. As a result, the final assembly structure is often governed by a delicate balance between molecule–molecule and molecule–substrate interactions, as well as by competitive adsorption and kinetic or thermodynamic effects, and therefore cannot always be readily inferred from the behavior of the individual components alone.^9,12,15^

Fullerene molecules, such as C_60_ and C_70_, typically assemble on metal surfaces through relatively weak van der Waals interactions, forming close-packed structures.^8,9,16,17^ Alkanethiols on Au(111) form self-assembled monolayers through chemisorption, giving rise to well-defined phases depending on coverage and thermal treatment. The combination of these two types of molecules therefore provides a platform to explore coassembly in systems with competing interaction mechanisms. We have recently studied the coassembly of alkanethiol and fullerene molecules on the Au(111) substrate^18–22^ under clean ultrahigh-vacuum conditions. Both alkanethiol and fullerene are mobile on Au(111) at room temperature, allowing efficient mixing of the molecules. The interaction between the two molecules is mainly of van der Waals in nature, with no specific functional groups involved. The lack of functional groups leads to a more versatile and fluidic interaction, characterized by high molecular mobility arising from weak, nondirectional interactions. The C_60_/octanethiol system, for instance, produces more than four distinct structures.^18,19^ Here, we report findings from the assembly of C_70_/octanethiol on Au(111). An octanethiol (OT) molecule can bridge two C_70_ molecules via van der Waals interaction, giving rise to a uniform C_70_/octanethiol mixture. Inside the C_70_/octanethiol mixture, we observed C_70_ molecules with two orientations: (i) C_70_ with its long axis perpendicular to the gold substrate and (ii) with its long axis parallel to the substrate. Having a single molecule being able to select one of two possible orientations offers more flexibility in two-component self-assembly. The rod-like OT molecule can also change its orientation from lying down to standing up in response to its local environment. With both C_70_ and OT being able to take multiple orientations, interesting self-assembled two-dimensional plastic crystalline structures^23,24^ are observed.

The shape of the building block in self-assembled structures is a very important parameter which controls efficient space filling. Packing of oval shapes in 3D, e.g., has no single universal solution. The packing scheme depends on many parameters such as the aspect ratio and orientation.^25–27^ An extensive effort is being made to develop new algorithms and apply computer simulations.^25,28^ The findings from this study have important implications and offer a different angle of view of the challenging problem in packing of nonspherical objects. We selected gold as the substrate to minimize the role played by the substrate and avoid the complete locking of OT or C_70_ by molecule–substrate interactions. Substrates such as graphite or certain types of semiconductors are more inert than gold in terms of thiol and C_70_/substrate interactions. However, Au(111) is a well-established system for both thiol and fullerene adsorption, with well-understood behavior. In this context, the molecule–substrate interaction is sufficiently strong to influence molecular packing and ordering, while still allowing molecular mobility at room temperature. The anchoring of the thiol molecules to Au(111) is important in this work. However, the anchoring is not strong and the “anchored” molecules can easily shift on the surface.

## Experimental Section

All STM measurements were performed in an Omicron low-temperature scanning tunneling microscope (LT-STM) system under ultrahigh-vacuum (UHV) conditions with a pressure better than 1 × 10^–10^ mbar. The Au(111) sample is a piece of (111) oriented single crystal with a purity of 99.99%. The sample is treated in a UHV system by many cycles of argon ion bombardment and thermal annealing at 800 K until the surface shows a clean herringbone reconstruction pattern.^5^ The sample is then taken out of the UHV system and immersed in a 1 mM octanethiol/ethanol solution for 24 h. Octanethiol (≥98.5%, Sigma-Aldrich) was used as received without further purification. This allows the formation of a dense layer of OT with a coverage of 1/3 monolayer (ML).^29^ 1.0 ML corresponds to the condition that for each Au atom on the surface, there is one OT molecule. We follow the same definition for the coverage of C_70_. The sample is then rinsed with pure ethanol to remove residues of OT and transferred back to the UHV chamber. Heating of the sample to 390 K is performed to partially desorb OT, leading to the formation of a lower-density striped OT phase. C_70_ molecules (purity 99%) are subsequently deposited via sublimation onto the striped phase under UHV conditions at room temperature (RT).

## Results with Discussion

Figure 1A shows a scanning tunnelling microscopy (STM) image of OT in the striped phase. The rows are along [112̅] directions. The perpendicular spacing between adjacent rows is *d* = 2.64 ± 0.02 nm. Figure 1B shows the corresponding molecular model, which consists of rows of RS–Au–SR “staples”,^30,31^ i.e., Au-adatom-dithiolate that make a three-point contact with the gold substrate via the Au adatom and the two sulfur atoms. The two SR chains are parallel to the substrate in this striped phase due to the optimization of van der Waals interaction between the molecule and the substrate. As will be shown later, adding C_70_ to the substrate forces the SR chains to stand up. The staple rows are parallel to the [112̅] direction with adjacent staples spaced at 0.5 nm (≈ $\sqrt{3a}$ ), where *a* is the nearest-neighbor Au–Au distance. Due to the 3-fold symmetry of the Au(111) surface, the staple rows are aligned with any one of three equivalent directions. We arbitrarily assign the row direction as [112̅]. Along the [11̅0] direction, the perpendicular distance *d* between two adjacent rows is 2.64 ± 0.02 nm, which is ∼9*a*. As the stripe direction is perpendicular to the close-packed direction of the Au(111) surface, the periodicity can be expressed in terms of *p*, defined as the number of nearest-neighbor Au–Au spacings along this direction.^32,33^ The observed spacing therefore corresponds to a *p* (9 × $\sqrt{3}$ ) phase, in which the staple units are separated by $\sqrt{3}a$ along the stripe direction. Thus, the corresponding coverage of OT is 0.13 ML. The OT chains are nearly parallel to the Au(111) substrate such that the Au(111) is effectively covered. At room temperature, the OT chains are not stationary; they flip up and down due to thermal energy.^1,34^ The interaction between the alkyl chains and the Au(111) substrate is dominated by weak van der Waals forces. The adsorption energy of *n*-alkanes on Au(111) has been reported to be approximately 6.2 kJ mol^–1^ per CH_2_ unit.^35,36^ This relatively weak interaction suggests that the alkyl chains are not rigidly locked to specific adsorption sites and can adopt different conformations within the monolayer.

**Figure 1:**
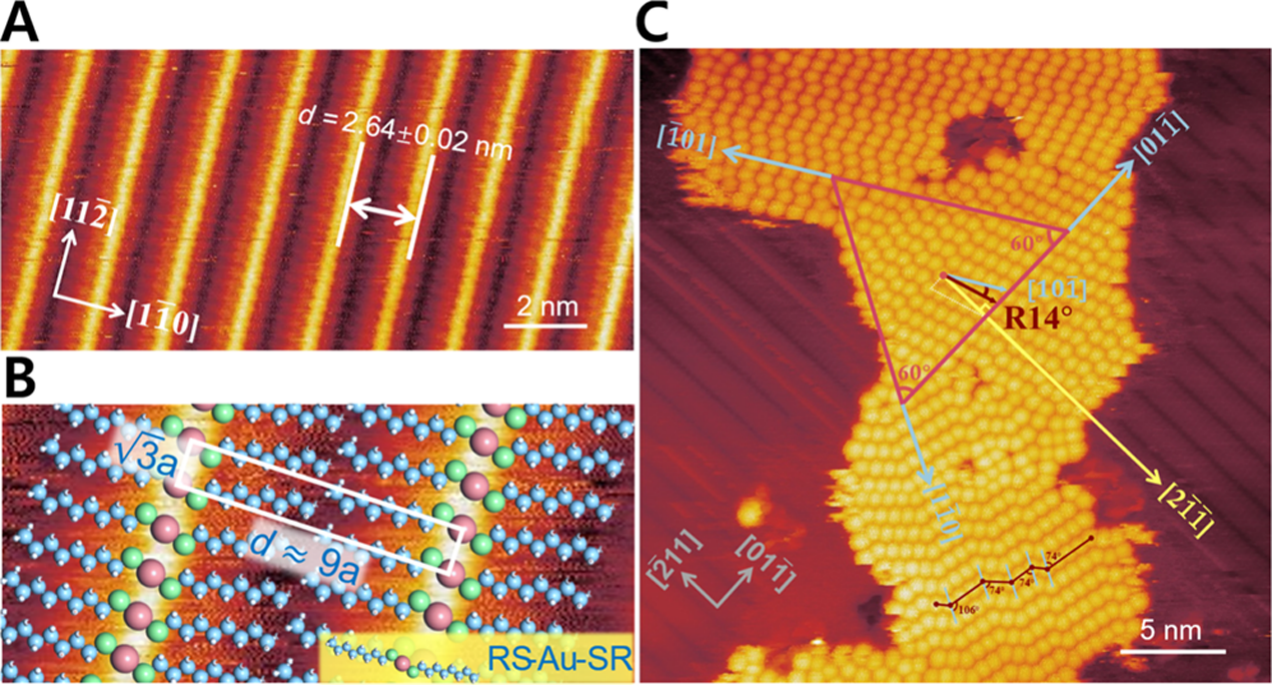
STM images of octanethiol and coassembled C_70_/OT mixture on Au(111). (A) STM image of the striped phase of octanethiol on Au(111) (*V*_b_ = 0.9 V, *I*_t_ = 150 pA). The rows are along the [112̅] direction. The perpendicular spacing between adjacent rows is *d* = 2.64 ± 0.02 nm. (B) Corresponding molecular model with the RS–Au–SR staples overlaid on the STM image. The green spheres are S atoms. The unit cell is indicated, the perpendicular spacing between adjacent rows *d* ≈ 9*a*, where *a* is the nearest-neighbor Au–Au distance. The staple units are separated by the $\sqrt{3}a$ along the stripe direction. (C) STM topographic image (*V*_b_ = 1.3 V, *I*_t_ = 180 pA) after subsequent deposition of C_70_ molecules onto the OT-covered Au(111) surface. The close-packed rows of C_70_ molecules are along the direction 14° from the [101̅] direction.

Figure 1C shows an STM image acquired 24 h after 0.05 ML of C_70_ molecules were deposited, by which time a regular C_70_/OT mixed structure had formed. Immediately after deposition, C_70_ is seen to mix with OT in a rather disordered amorphous layer with the staple rows completely disrupted. The interaction between C_70_ and OT prevents the formation of 2D close-packed C_70_ on the surface. In Figure 1C, we can see short segments of C_70_ molecules with an intermolecular distance of 1.03 nm. The longest segment consists of six C_70_ molecules, while the shortest segment consists of just two molecules. The C_70_ segments do not follow the [112̅] direction; they are 14 degrees off the [101̅] direction. This is very similar to what is observed for the R14 phases of C_60_ ^16^ in the absence of OT, where fullerene molecules form close-packed rows along the direction 14 degrees off [101̅]. Although such substrate-locked phases have not been identified for C_70_, its adsorption is likewise governed by relatively weak molecule–substrate interactions, leading to a greater degree of orientational freedom. The gap between the C_70_ rows is filled by OT molecules, as will be shown with high-resolution images later. Without OT molecules, C_70_ would all condense into a close-packed structure.^17^ Next to the C_70_/OT mixture in Figure 1C, one can see the existence of OT-only regions, suggesting that individual C_70_ molecules are mobile after landing, and a stable C_70_/OT nucleus is required prior to the formation of an extended 2D C_70_/OT mixture.

Figure 2A,B presents STM images acquired from the same surface area with a tunnelling current of 50 pA at positive (+1.4 V) and negative (−1.2 V) sample bias, respectively. Some molecules are clearly seen as ovals, while others appear more circular. Figure 2C displays height profiles taken along a row of oval molecules (line D in Figure 2A) and a row of circular molecules (line L in Figure 2A) under positive sample bias. Figure 2D shows the height profiles for the same two rows of molecules under negative sample bias. The height profile shows that the circular-shaped molecules are 0.09 nm taller than the oval ones (Figure 2C,D). This height difference does not change when the polarity of the bias is switched from positive to negative. Therefore, this apparent height difference is mainly contributed by a difference in the physical height of the molecules. The value of 0.09 nm is consistent with the difference between a standing-up C_70_ molecule and a lying-down molecule.

**Figure 2:**
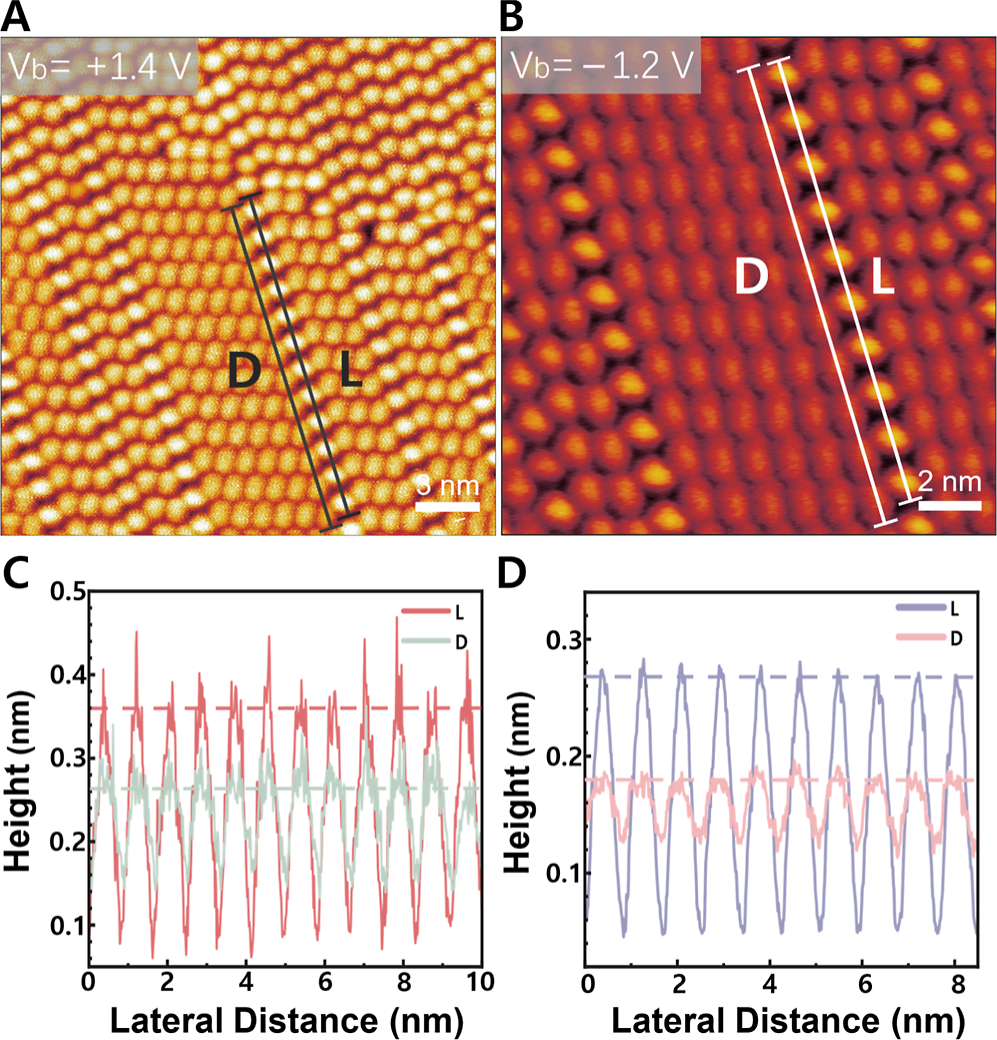
STM images acquired at different sample biases and the corresponding height profiles along C_70_ rows. (A,B) Comparison between STM images acquired at *V*_b_ = +1.4 V (A) and *V*_b_ = −1.2 V (B), with a tunneling current of 50 pA. In both images, “brighter” C_70_ molecules can be observed. One bright row “L” and a dark row “D” are highlighted in (A,B), respectively. Corresponding height profiles along “L” and “D” are displayed in (C,D).

At RT, the C_70_ molecules have some freedom to rotate or flip. This prevented the observation of the internal structure of the molecules. Furthermore, significant thermal motion of the OT chains results in the STM being unable to resolve the OT molecules. Imaging at 77 K overcomes all of these difficulties by freezing the molecular motion. Figure 3 shows STM images acquired at 77 K. While the global structure of the 2D OT/C_70_ layer remains essentially unchanged upon cooling to 77 K, the molecular orientations can now be determined from the internal structure of the molecules revealed by STM. Between the C_70_ rows, signals from OT molecules are also observed. However, the features from OT molecules are highly sensitive to the bias voltage and are best resolved at 1.6 V. The instability of the tunnel signal from the OT is likely due to tip-induced movement of the alkane chain. For C_70_, the three most observed orientations are marked by rectangular boxes labeled as #1, #2, and #3 in Figure 3A. The dashed line marks a boundary between two structural domains (I and II). In comparison with region II, region I exhibits a higher occurrence of C_70_ with orientation #3. Figure 3B shows a high-resolution STM image of another area with the same structure as region II in Figure 3A. All features in Figure 3B are labeled with different colored shapes. Statistics based on simple counting reveal that orientation #1 (marked with white circles) is the most prevalent adsorption configuration in this region. Yellow circles mark the observed features from OT, although not all OT molecules are observed. Some OT molecules may have their alkane chains heavily tilted away from the surface normal and are thus too far from the tip to be seen. The statistical distribution of the different adsorption configurations is summarized in the pie chart shown in Figure 3C. The analysis is based on the representative STM image in Figure 3B, from which the occurrences of each configuration were counted. The results confirm that orientation 1 is the dominant configuration, while orientations 2 and 3 appear with lower probabilities. Although the analysis is based on a limited data set, the observed distribution provides a clear indication of the predominant adsorption configurations in this system.

**Figure 3:**
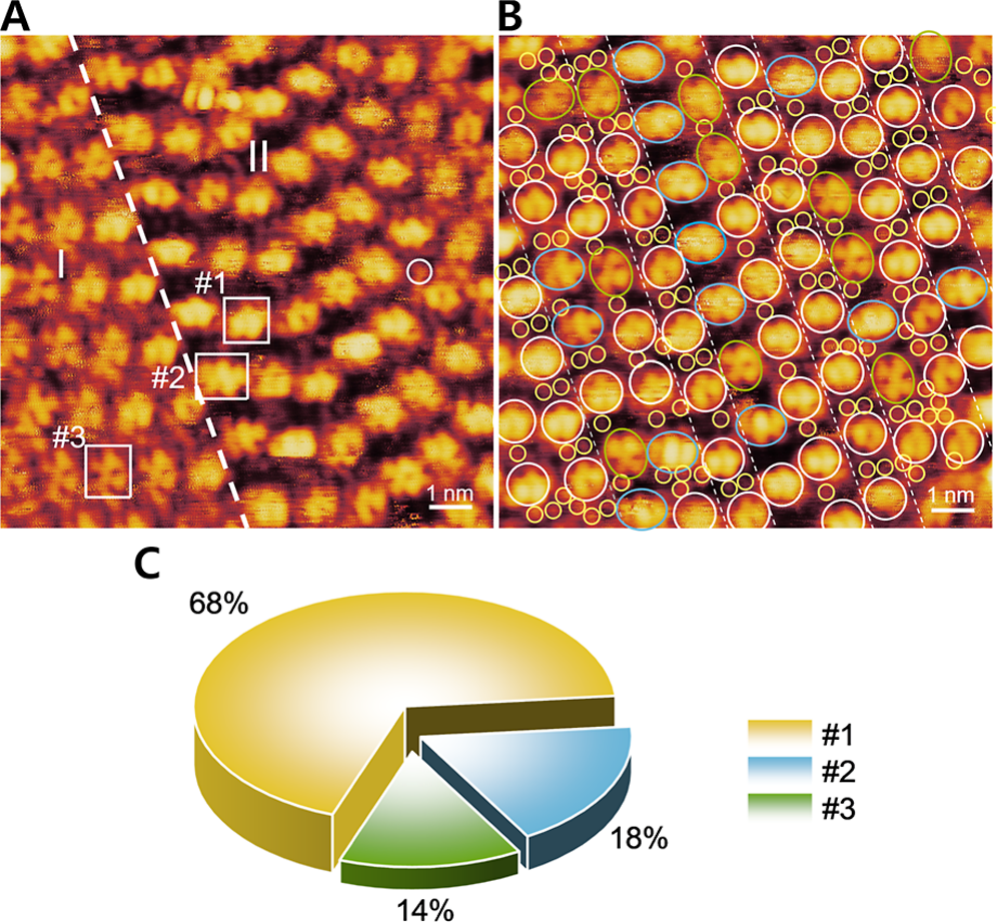
High-resolution STM images of mixed C_70_–OT acquired at 77 K. (A) STM topographic image (*V*_b_ = 1.6 V, *I*_t_ = 100 pA) of mixed C_70_–OT on Au(111) acquired at 77 K. Three most frequently observed orientations of C_70_ molecules are marked with rectangular boxes and labeled as #1, #2, and #3. The white circle marks one of the features that arise from OT. The dashed line highlights the boundary joining two structural domains. (B) STM image acquired under identical imaging conditions (*V*_b_ = 1.6 V, *I*_t_ = 100 pA) from a different nearby region of the same sample. White circles mark C_70_ molecules with orientation #1. Blue ovals mark molecules with orientation #2. Green ovals mark molecules with orientation #3. Small yellow circles mark the locations where individual alkane chains are observed. (C) The statistical distribution of the adsorption configurations based on Figure 3B is summarized in the pie chart, showing that configuration #1 accounts for 68% of the observed molecules, while configurations #2 and #3 account for 18% and 14%, respectively.

High-resolution STM images such as the ones shown in Figure 3 provide accurate information about the relative locations of the C_70_ molecules as well as their orientations. In order to determine the structure of the mixed C_70_–OT, we first place the C_70_ molecules on a close-packed Au(111) surface guided by the images. We then fill the gaps with the OT staples with the constraint that the Au-adatom sits on a bridge site. The proposed structural model is intended to represent the relative arrangement and spacing of the C_70_ molecules, which are well resolved in the STM images. Since the STM image shows only the tail of the alkane chain, trial and error is applied to optimize the adsorption site for each staple. The proposed structural model shown in Figure 4 is a good representation of the C_70_-OT layer, and it forms a useful starting point for further computerized optimization. Figure 4A–C shows the three most frequently observed orientations of C_70_. Corresponding to the three experimental images of the upper panels, the lower panels present the adsorption configurations. Type #1 corresponds to C_70_ with a pentagonal ring facing upward on the Au(111) surface (Figure 4A). Type #2 and type #3 present two distinct adsorption orientations of flat-lying C_70_ molecules: one with a carbon atom facing upward (Figure 4B), and the other with the hexagon–hexagon face facing upward, denoted as 6:6 ring-up (Figure 4C).^37,38^ The signal from the space not occupied by the C_70_ molecule is attributed to OT. At RT, no signal from OT can be detected due to the rapid thermal motion of the alkane chains. We expect to see alkane chains appearing in pairs. However, we observed many unpaired chains. Since there is always a sizable open space next to an unpaired chain, we conclude that the apparent unpaired chain is from a staple with one of its chains tilted too far away from the surface normal and hence not detected by STM. In Figure 4F, we have not included the “invisible” tilted chains.

**Figure 4:**
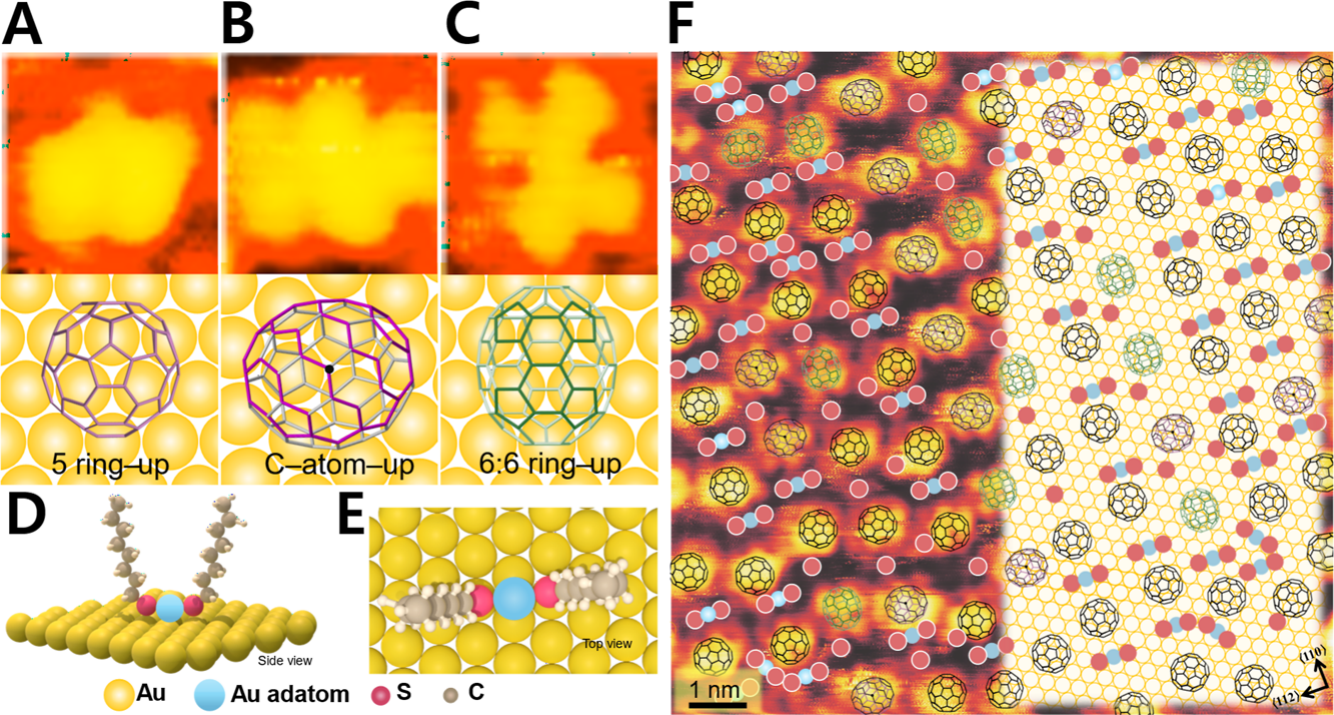
Proposed structural model of the C_70_–OT mixture on the Au(111) surface. (A–C) Three most frequently observed adsorption orientations of C_70_ molecules on Au(111) observed by STM. The upper panels are experimental images. The corresponding molecular orientations are shown in the lower panels: 5 ring-up, carbon-atom-up, and 6:6 ring-up. (D) The alkane chains of the RS–Au–SR “staples” tend to stand upright, occupying the space between C_70_ molecules. Each alkane chain bridges two C_70_ molecules. (E) Top view of the RS–Au–SR “staples”. The Au adatom sits at the bridge site on Au(111). The S atom sits directly above a Au atom. (F) Structural model overlaid on top of the STM image, which is taken from Figure 3B. The right half of the image is completely replaced by the structural model so that one can see the relationship between the molecules and the top layer of Au atoms. The alkane chains should appear in pairs, as expected from the staple motif. However, nonpaired chains can be seen in (F) because some chains do not appear in the STM image.

During the construction of the structural model, we encountered a problem when trying to fill the space between C_70_ molecules with OT staples. It is not possible to place the staples such that a staple is at the same distance from the neighboring C_70_ molecules. Optimization of the van der Waals interaction favors the configuration where an OT chain bridges two C_70_ molecules, with the OT chain halfway between the two bridged C_70_ molecules. However, this seems to be impossible if we strictly keep the staple at its preferred bonding site on Au(111). There is thus the competition between the preferred adsorption site for the staple on Au(111) and the preferred distance between the OT chain and its nearest neighbor C_70_ molecules. In STM images obtained at 77 K, we can see that some OT chains are slightly skewed toward C_70_ molecules on one side. It is possible that at RT, the dynamic movement of the OT chain allows the chain to interact with C_70_ molecules on either side. On cooling to 77 K, the OT chain loses its thermal motion and becomes tilted toward one of the two C_70_ molecules. Figure 4F shows the top view of the proposed structural model, in which the Au adatom of the staple occupies the bridge sites on Au(111). Although the alkane chains are mostly standing perpendicular to the substrate, there are OT chains with an unknown tilt angle. Figure 4F is based on the STM image of Figure 3B, which has been divided into two halves: the left half keeps the original image, while the right half is modified by replacing the C_70_ molecules in the image with wire frame models of the molecules. The Au atoms in the Au(111) substrate are included in the right half of the figure to show the relative positions of C_70_ and OT.

It is a common phenomenon that two-component materials such as metallic alloys exhibit different phases controlled by the number ratio of the two components. For the fullerene/alkanethiol system, the fullerene/thiol ratio does indeed control the structural phases.^18–22^ Here, with C_70_ and OT, it is likely that the starting C_70_/OT ratio favors the formation of the region I structure with flat-lying C_70_ molecules. In Figure 3, we see rather regular arrays of flat-lying C_70_ molecules. However, the arrays of flat-lying C_70_ are frequently interrupted by a row of standing-up C_70_. Since the standing-up C_70_ has a smaller footprint, we suspect that the standing-up process serves the purpose of accommodation excessive number of thiol molecules. A possible reaction pathway is the formation of the regular array of flat-lying C_70_ molecules with OT incorporated. A small number of OT are released in that process, thus increasing the OT/C_70_ ratio. To compensate for this changing ratio, C_70_ starts to stand up, absorbing the excess number of OT as a result.

The high-resolution images at 77 K show relatively good translational order of the C_70_ molecules within each domain. The orientational order is rather poor, even within the same domain. The structure of the C_70_/OT mixture follows more or less that of a plastic crystal. A fundamental reason for the formation of such a two-dimensional plastic crystal of C_70_/OT is that the conditions for both C_70_ and OT to occupy their preferred adsorption sites while maintaining an effective van der Waals interaction cannot be met. The two molecules have their own choices of adsorption sites, and that does not meet the requirement for optimized interaction between the alkane chain and the C_70_ molecules. The extra degree of freedom of the C_70_ molecule for being able to switch its orientation, plus the degrees of freedom of the OT chain to adjust its tilt angle, are believed to play a key role in forming a two-dimensional plastic crystalline structure. According to the structural model of Figure 4F, the coverage of OT is 0.1 ML, corresponding to an OT/C_70_ ratio of 2:1. The starting coverage of OT before the deposition of C_70_ is 0.13 ML, which is more than that required to form the OT/C_70_ mixture. With some C_70_ molecules taking the standing-up orientation, a maximum amount of OT from the 0.13 ML is incorporated into the bicomponent mixture. The OT/C_70_ mixture shows the character of a plastic-crystal phase that does not have a long-range orientational order. The translational order is also limited to just a few nanometers. For charged colloidal particles, orientational ordering has been achieved by the influence of an applied electric field.^39,40^ For fullerene molecules, the electric field effect is limited unless an extremely high field is applied, causing the molecules to significantly polarize. We plan to look at this effect by inducing polarization with the STM tip.

## Conclusion

In summary, this study investigates the self-assembly behavior of C_70_ fullerenes in the presence of octanethiol on a Au(111) surface. Using surface-sensitive characterization techniques, the results show that C_70_ molecules can adopt two distinct adsorption geometries: a standing-up orientation with the long molecular axis perpendicular to the surface and a lying-down orientation with the axis parallel to the surface. The coexistence of these orientations significantly enhances the structural flexibility of the molecular overlayers. As a consequence, the system is able to form a variety of ordered arrangements, including two-dimensional structures that exhibit characteristics analogous to those of plastic crystals, where positional order is maintained while orientational degrees of freedom remain partially disordered. These findings highlight how molecular anisotropy and coadsorbate interactions can be exploited to tune surface assemblies and engineer functional, flexible molecular architectures on metal substrates.^41^
